# Rutin alleviated acrolein-induced cytotoxicity in Caco-2 and GES-1 cells by forming a cyclic hemiacetal product

**DOI:** 10.3389/fnut.2022.976400

**Published:** 2022-08-16

**Authors:** Peifang Chen, Shuang Liu, Zhao Yin, Pengjie Liang, Chunhua Wang, Hanyue Zhu, Yang Liu, Shiyi Ou, Guoqiang Li

**Affiliations:** ^1^Department of Food Science, Foshan University, Foshan, China; ^2^Department of Hematology, Guangdong Second Provincial General Hospital, Guangzhou, China; ^3^Department of Food Science and Engineering, Jinan University, Guangzhou, China; ^4^Guangdong Provincial Key Laboratory of Intelligent Food Manufacturing, Foshan University, Foshan, China; ^5^South China National Center for Food Safety Research and Development, Foshan University, Foshan, China

**Keywords:** acrolein, rutin, hemiacetal adduct, simulated *in vitro* digestion, cytotoxicity

## Abstract

Acrolein (ACR), an α, β-unsaturated aldehyde, is a toxic compound formed during food processing, and the use of phenolics derived from dietary materials to scavenge ACR is a hot spot. In this study, rutin, a polyphenol widely present in various dietary materials, was used to investigate its capacity to scavenge ACR. It was shown that more than 98% of ACR was eliminated under the conditions of reaction time of 2 h, temperature of 80 °C, and molar ratio of rutin/ACR of 2/1. Further structural characterization of the formed adduct revealed that the adduct of rutin to ACR to form a cyclic hemiacetal compound (RAC) was the main scavenging mechanism. Besides, the stability of RAC during simulated *in vitro* digestion was evaluated, which showed that more than 83.61% of RAC was remained. Furthermore, the cytotoxicity of RAC against Caco-2 and GES-1 cells was significantly reduced compared with ACR, where the IC_50_ values of ACR were both below 20 μM while that of RAC were both above 140 μM. And the improvement of the loss of mitochondrial membrane potential (MMP) by RAC might be one of the detoxification pathways. The present study indicated that rutin was one of the potential ACR scavengers among natural polyphenols.

## Introduction

Acrolein (ACR), an α, β-unsaturated aldehyde, is one of the typical active carbonyl compounds (1). ACR could be produced endogenously by enzyme-mediated metabolism of threonine and polyamines, metabolism of the anticancer drug cyclophosphamide, and oxidation of unsaturated fatty acids on the cell membrane (2–5). Due to the presence of the olefinic double bond and carbonyl group, ACR could react with biological nucleophiles such as DNA and proteins, resulting in dysfunction of biomacromolecules (6). Besides, ACR mediated cytotoxicity and genotoxicity through mitochondrial dysfunction, oxidative stress, endoplasmic reticulum stress and so on (7–9). It was also reported that ACR was associated with several diseases, including chronic kidney disease, cardiovascular disease, and Alzheimer's disease (AD) (10–12). However, ACR could be metabolized into *S*-(hydroxypropyl)-mercapturic acid (3-HPMA) *in vivo* as an elimination pathway (13). And the deregulated ACR metabolism to generate 3-HPMA was found in AD patients (14, 15). On the other side, ACR was formed exogenously. The combustion of organic stuff, for instance, gasoline, plastics, and cigarettes, resulted in the presence of ACR in the environment (16, 17). Notably, ACR was even found in foodstuffs, such as cheese, donuts, beer, and wine, ranging from 10 to 600 μg/kg (12, 13, 18). And vegetable oil rich in polyunsaturated fatty acids was another important source of ACR, which could generate ACR through oxidative degradation under high-temperature treatment. It was reported that after heating at 180°C for 24 h the content of ACR reached 29.3 mg/kg in olive oil, 156.4 mg/kg in rapeseed oil, and 207.4 mg/kg in linseed oil (19). Though endogenous ACR could be scavenged by enzymatic metabolism, a large amount of of exogenous ACR poses a serious threat to human health.

In recent years, searching for naturally occurring polyphenols to effectively capture ACR has received extensive attention (20). A study performed by Zhu et al. (21) investigated the capacity of 21 natural polyphenols to trap ACR under simulated physiological conditions and found that phloretin exhibited an outstanding ability to scavenge ACR, with more than 99% of ACR being quenched. After that, other polyphenols, such as myricetin, curcumin, and tea catechins, were also shown to remove this detrimental compound (22–25). In fact, it was the *meta*-phenol structure of polyphenols that played an essential role in their reaction with ACR (26). Inspired by previous literature, we paid more attention to potential ACR scavengers derived from dietary materials.

Previously, a study by Zamora et al. (27) showed that ACR was trapped by quercetin to form an equimolar adduct that was detected not only in an experimental model of frying onions with fresh rapeseed oil but also in the commercial crispy fried onions. While quercetin is the dominant flavonol in onions, it usually exists in the form of glycosides, such as rutin (quercetin 3-*O*-rhamnosylglucoside) (28, 29). Literature also reported that rutin was the second most abundant flavonoid in dry red onion skins after quercetin (30). Besides, rutin is also widely present in asparagus, buckwheat, and peppers, being an important dietary constituent (31–33). In addition to culinary virtues, substantial evidence suggested that rutin was involved in a variety of biological activities, including antioxidant, antiallergic, anti-inflammatory, and cardioprotective effects (34, 35). Chemically, rutin also possesses the typical *meta*-phenol structure as a reaction site for ACR, but the scavenging effect of rutin on ACR was lack of discussion.

In this work, a simulated system was established to investigate the effect of three factors on the scavenging of ACR by rutin. Subsequently, the adduct between ACR and rutin was identified, which also helped to elucidate the reaction mechanism. Considering that the adduct might be formed and ingested after cooking, a three-stage simulated *in vitro* digestion of the adduct was conducted. Furthermore, the cytotoxicity of RAC against the human intestinal epithelial cells (Caco-2) and gastric epithelial cells (GES-1) was determined with ACR as the control, followed by the measurement of mitochondrial membrane potential (MMP).

## Materials and methods

### Materials and chemicals

Acrolein (95%) was acquired from Energy Chemical Co., Ltd. Rutin (98%), 2,4-dinitrophenylhydrazine (DNPH), and ACR-DNPH standard were from Shanghai Macklin Biochemical Co., Ltd. Dulbecco's Modified Eagle Medium (DMEM), Fetal Bovine Serum (FBS), non-essential amino acids (NEAA), penicillin, streptomycin, trypsin-EDTA, and phosphate buffered saline (PBS, pH = 7.4) were bought from Thermo Fisher Scientific Inc. (MA, USA). Simulated saliva (SSF), gastric (SGF), and intestinal (SIF) fluids were from Phygene Biotechnology Co., Ltd. (Fuzhou, China). Cell counting kit-8 (CCK-8), apoptosis detection kit (including Annexin-V fluorescein isothiocyanate, Annexin-V FITC, and propidium iodide, PI), and JC-1 mitochondrial membrane potential assay kit were from Shanghai BestBio Co., Ltd. All the analytical and high-performance liquid chromatography (HPLC) grade reagents were obtained from Sigma-Aldrich (St. Louis, MO, USA). Sephadex^TM^ LH-20 column fillers were obtained from GE Healthcare Bio-Sciences AB (Uppsala, Sweden).

### The influence of temperature, reaction time, and molar ratio on the elimination of ACR by rutin

In the present study, three variables were taken into consideration, namely temperature (40–100°C), reaction duration (0.5–8 h), and molar ratio of rutin to ACR (from 1/2 to 4/1), and the remaining level of ACR was determined. A mixture of rutin, ACR, and 20 mL of PBS was prepared in a 50 mL three-necked flask and reacted under light-proof conditions. The different variables were studied separately while keeping other conditions constant as follows: temperature of 80°C, reaction time of 2 h, and the molar ratio of rutin/ACR of 1/1. At the end of each reaction, ACR was derivatized with DNPH, and its content was determined as below.

### Qualitative and quantitative determination of ACR

The stock solution equivalent to ACR concentration of 100 mg/mL was prepared by dissolving 10.7 mg of ACR-DNPH standard with 25 mL of acetonitrile (ACN). And the ACR-DNPH standard curve (2 to 100 μg/mL) was established by serial dilutions of the stock solution. The HPLC conditions were as below. An Agilent 1,260 Infinity II HPLC system (Agilent Technologies, Palo Alto, CA) equipped with a DAD and a Zorbax SB-C18 column (250 × 4.6 mm; 5 μm; Agilent Technologies) was utilized for the determination of ACR-DNPH. The separation was conducted under isocratic conditions by introducing 50% A (water) and 50% B (ACN) at a flow rate of 1.0 mL/min for 15 min. The temperature was 35°C, the injection volume was 20 μL, and the detection wavelength was set at 365 nm. According to the retention time as well as the peak area, the standard curve of ACR-DNPH derivates was obtained.

DNPH was recrystallized as the followings before use: excess DNPH was added into 200 mL of methanol (MeOH) heating for 1 h, and the supernatant was collected and further heated at 60°C to remove 95% of the solvent; the crystals were then washed twice with 100 mL of MeOH, transferred to another clean beaker, and co-heated with 200 mL of MeOH; and the resulting crystal was stored under seal. The 0.1 M DNPH solution was prepared by dissolving 5.0548 g of the purified DNPH in 250 mL of ACN containing 5 mL of 10% phosphoric acid solution.

Derivatization of ACR with DNPH was conducted in accordance with Reilly et al. (36). In short, one milliliter of each sample was incubated with 1 mL of diluted DNPH solution (0.01 M) for 40 min at 40°C, followed by extraction with 10 mL of MeOH/water (75: 25, v/v). After centrifugation (6,000 rpm, 10 min), the supernatant was collected and was further extracted with 10 mL of dichloromethane to obtain the DNPH derivatives. Prior to HPLC analysis as above, the DNPH derivatives were re-dissolved in 1 mL of HPLC grade ACN and passed through a 0.45 μm filter (Bie & Berntsen, Rødovre, Denmark).

### Preparation and purification of the rutin-acrolein adduct (RAC)

Based on the above results, rutin (2 mmol) and ACR (1 mmol) dissolved in 25 mL of PBS were mixed in a three-necked flask and reacted at 80°C for 2 h in the dark. After cooling to room temperature, the supernatant was obtained by vacuum filtration and concentrated to 2 mL in a rotary evaporator at 45°C before loading onto a Sephadex LH-20 column (105 × 2.2 cm) for purification. MeOH was applied as the eluent and the flow rate was set at 0.6 mL/min. As a result, the purified adduct of rutin and ACR, namely RAC, was obtained.

### Structural characterization of the adduct

The reaction progress was tracked and determined by the Shimadzu LC-20 system (Tokyo, Japan) equipped with a diode array detector (DAD). The HPLC conditions were as follows: the mobile phase was composed of MeOH (A) and 0.1% acetic acid water (B) with gradient elution from 10% A to 100% A within 30 min at a flow rate of 1.0 mL/min. With an injection volume of 10 μL, a Zorbax SB-C18 column (150 × 4.6 mm; 5 μm; Agilent Technologies) was used and the UV spectra were recorded in the range of 190–400 nm.

The mass spectrum (MS) data was obtained in the negative mode by direct injection into an LCMS-8045 triple quadrupole mass spectrometer (Shimadzu Corporation, Tokyo, Japan) equipped with electrospray as the ionization source. The scan range was from *m*/*z* 50 to *m*/*z* 1000. Other parameters for MS and MS/MS measurements were set in refer to the method of Qi et al. (37).

Additionally, 0.5 mL of DMSO-*d*_6_ and 15 mg of the sample were added into a 5-mm nuclear magnetic resonance (NMR) tube, and the spectra, including ^1^H NMR, ^13^C NMR, distortionless enhancement by polarization transfer 135 (DEPT 135), ^1^H-^1^H correlation spectroscopy (COSY), heteronuclear single-quantum correlation (HSQC) and heteronuclear multiple-bond correlation (HMBC), were recorded on a Bruker 600 MHz NMR apparatus (Bruker Corp, Fallanden, Switzerland). Rutin was applied as the reference.

Rutin. ^1^H NMR (600 MHz, DMSO-*d*_6_): δ 12.60 (s, 1H, 5-OH), 10.84 (s, 1H, 7-OH), 9.67 (s, 1H, 4'-OH), 9.18 (s, 1H, 3'-OH), 7.55 (m, 1H, H-6'), 7.53 (s, 1H, H-5'), 6.84 (d, *J* = 8.3 Hz, 1H, H-2'), 6.39 (d, *J* = 2.0 Hz, 1H, H-8), 6.19 (d, *J* = 2.0 Hz, 1H, H-6), 5.34 (d, *J* = 7.1 Hz, 1H, H-1 Glc), 4.38 (s, 1H, H-1 Rha), 3.05–3.72 (m, protons for glycoside region), 0.99 (d, *J* = 6.2 Hz, 3H, H-6 Rha). ^13^C NMR (150 MHz, DMSO-*d*_6_): δ 177.4 (C-4), 164.0 (C-7), 161.2 (C-5), 156.6 (C-2), 156.4 (C-9), 148.4 (C-4'), 144.7 (C-3'), 133.3 (C-3), 121.6 (C-6'), 121.2 (C-1'), 116.3 (C-5'), 115.2 (C-2'), 104.0 (C-10), 101.2 (Glc, C-1”), 100.7 (Rha, C-1”'), 98.7 (C-6), 93.6 (C-8), 76.5 (Glc, C-3”), 75.9 (Glc, C-5”), 74.1 (Glc, C-2”), 71.8 (Rha, C-4”'), 70.6 (Rha, C-3”'), 70.4 (Rha, C-2”'), 70.0 (Glc, C-4”), 68.2 (Rha, C-5”'), 67.0 (Glc, C-6”), 17.7 (Rha, C-6”'). The obtained NMR data showed consistence with those in the literature (38).

### *In vitro* simulated digestion

The simulated oral, gastric, and intestinal digestion experiments of RAC were carried out according to Mamone et al. (39) with minor adjustments. One milliliter of RAC (1 mg/mL) was mixed with 4 mL SSF and stirred at 170 rpm for 2 min in lightproof condition to mimic the oral phase digestion. Based on the oral stage, 10 mL of SGF was added and reacted for another 2 h under the same condition to imitate the gastric phase digestion. To investigate the intestinal phase digestion, 20 mL of SIF was further added, and the mixture was stirred for another 2 h after simulated gastric digestion. For each stage, the same volume of deionized water was used in place of the corresponding digestive fluid as the control. At the end of each stage, 0.8 mL of the mixture was withdrawn and diluted with MeOH to 1.0 mL for HPLC analysis.

The standard curve of RAC was prepared as follows: one milligram of RAC was dissolved with MeOH to a final concentration of 1 mg/mL. The above solution was further used to prepare a series of RAC concentrations at 0.02, 0.04, 0.08, 0.2, and 1.0 mg/mL, respectively. The HPLC conditions for the determination of the content of RAC in the digestion mixture and the preparation of the standard curve of RAC were the same as those for the analysis of RAC, except that the detection wavelength was at 358 nm.

### Cell culture

The Caco-2 cell line and GES-1 cell line were obtained from iCell Bioscience (Shanghai, China). As described by Chai et al. (40) with a bit modification, the Caco-2 cells were cultured within a medium composed of DMEM supplemented with 1% (v/v) NEAA, 10% (v/v) FBS, and penicillin and streptomycin (100 U/mL). GES-1 cells were cultivated according to Xu et al. (41) with slight modifications as follows: DMEM medium including 1% (v/v) NEAA, 10% (v/v) FBS, and penicillin and streptomycin (100 U/mL). Cells were all maintained in a humidified incubator containing 5% CO_2_ and 95% air at 37°C.

### Cell survival

The effects of ACR and RAC on cell survival (both Caco-2 and GES-1 cells) were measured by the CCK-8 assay (41). Briefly, cells (1 × 10^4^ cells/well) were seeded into a 96-well plate and cultivated overnight. Then 100 μL of sample solutions at different concentrations were added into the wells, followed by incubation at 37°C for another 48 h. After cells were rinsed twice with PBS and resuspended in 100 μL of DMEM supplemented with 10% FBS, 10 μL of CCK-8 solution was added into each well and the plate was maintained for 1 h in the incubator. The plate was read at 450 nm on a microplate reader (BioTek, Epoch 2, USA), and all experiments were performed thrice. Cell viability was expressed as a percentage of the control group as shown below:


$$\begin{array}{l} \text{Cell viability }\left(\text{\%}\right)\;=\;A{\;}_{sample}/A{\;}_{control}\;\times \;100\text{\%} \end{array}$$

Where *A*_*sample*_ was the absorbance (Abs) of the cells incubated with different concentrations of sample solutions, and *A*_*control*_ was the Abs of the cells incubated without sample solution.

### Apoptosis assay

Cell apoptosis assay was conducted in refer to Abas et al. (42) with modifications. In short, cells at a density of 1 × 10^4^ cells/well were seeded in 96-well plates and incubated overnight, followed by exposure to different concentrations of ACR and RAC for another 24 h. After that, cells were rinsed twice with PBS, resuspended in 200 μL of Annexin-binding buffer, and finally stained with Annexin-V FITC and PI (5 μL each) for 10 min at 37°C in darkness. After adding 400 μL of Annexin-binding buffer, the signal intensity was measured using a FACSCalibur (Becton Dickinson, CA, USA) flow cytometry within 1 h, and data was analyzed with FlowJo software (Ashland, OR, USA).

### JC-1 staining

After incubation with different concentrations of samples, the changes of MMP in Caco-2 and GES-1 cells were measured in accordance with Liu et al., with slight modifications (43). In brief, cells (1 × 10^4^ cells/well) were exposed to different concentrations of ACR and RAC for 24 h, respectively. Then 500 μL of JC-1 staining solution was added into each well and the plate was further incubated at 37°C for 15 min. Cells were then rinsed twice with 1 mL of staining buffer and resuspended with 500 μL of staining buffer prior to MMP determination in the flow cytometry.

### Statistical analysis

All experiments were carried out in triplicate, and the data was expressed as mean ± standard deviation. As for statistical analysis, analysis of variance (ANOVA) was performed with GraphPad Prism version 9.0.0 (GraphPad Software Inc., San Diego, CA). Differences were considered significant if *p* < 0.05.

## Results and discussion

### Effects of different parameters on the elimination of ACR

**Figure 1:**
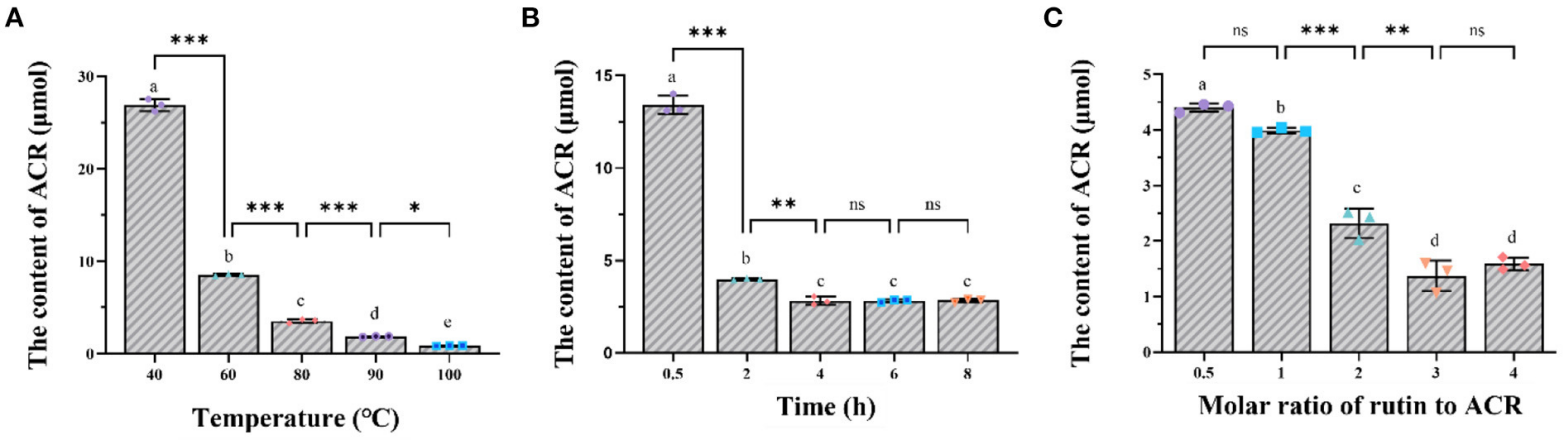
The impact of **(A)** temperature, **(B)** reaction time, and **(C)** molar ratio of rutin to ACR on the elimination of ACR by rutin. *: *p* < 0.05; **: *p* < 0.01; ***: *p* < 0.001; ns, not significant.

Based on the calibration curve (*y* = 326781*x*- 183802; *r*^2^ = 0.9999; *x* was the concentration of each sample and *y* was the corresponding peak area), the residual content of ACR was depicted in Figure 1. After incubation at 40, 60, 80, 90, and 100°C for 2 h, the content of ACR was determined to be 113.28, 35.9, 14.88, 7.83, and 3.60 μmol, respectively (Figure 1A). In other words, compared with the initial content (200 μmol), the amount of ACR was reduced by 43.36, 82.05, 92.56, 96.09, and 98.20%, respectively. A sharp decline of the content of ACR could be observed with the increase of temperature, especially from 40 to 80°C (*p* < 0.001), which indicated that temperature had a significant effect on the elimination of ACR (*p* < 0.05). Similar phenomena could be seen in regard to the effect of reaction time on the capture of ACR. As displayed in Figure 1B, ACR was eliminated by rutin in a time-dependent manner, with more than 94% being removed after 8 h of reaction. However, the content of ACR did not decrease significantly after 4 h of reaction (*p* > 0.05). Figure 1C showed the effect of molar ratio of rutin to ACR on the elimination of ACR. With the rise of molar ratio of rutin/ACR, the content of residual ACR exhibited an overall downward trend, from 18.54 μmol of 1/2 to 6.69 μmol of 4/1. Additionally, it could be observed that the content of ACR reduced significantly between the molar ratios of 1/1 and 2/1 (*p* < 0.001).

In addition to rutin, other phenolic compounds with a *meta*-phenol configuration in A ring were also found to capture ACR. According to Zhu et al. (21), after polyphenols (1.0 mM) incubating with ACR (0.5 mM) for 1.5 h at 37°C, the ability of them to remove ACR in a decrease sequence was phloretin, epigallocatechin-3-gallate, epicatechin-3-gallate, epicatechin, epigallocatechin, theaflavin-3,3'-digallate, theaflavin, cyanomaclurin, and phloridzin. Additionally, in the study by Wang et al. (25), resveratrol and hesperetin could eliminate 93.6 and 94.87% of ACR, respectively, after incubating with equal concentrations of ACR for 12 h at 37°C. Combined the above results, it was found that not only the specific structure of polyphenols but also the elevated temperature contributed to the higher scavenging efficiency of ACR. As a result, more RAC were prepared under the reaction conditions of temperature at 80°C, reaction time of 2 h, and molar ratio of rutin/ACR of 2/1 for subsequent experiments.

### Structural characterization of RAC

**Figure 2:**
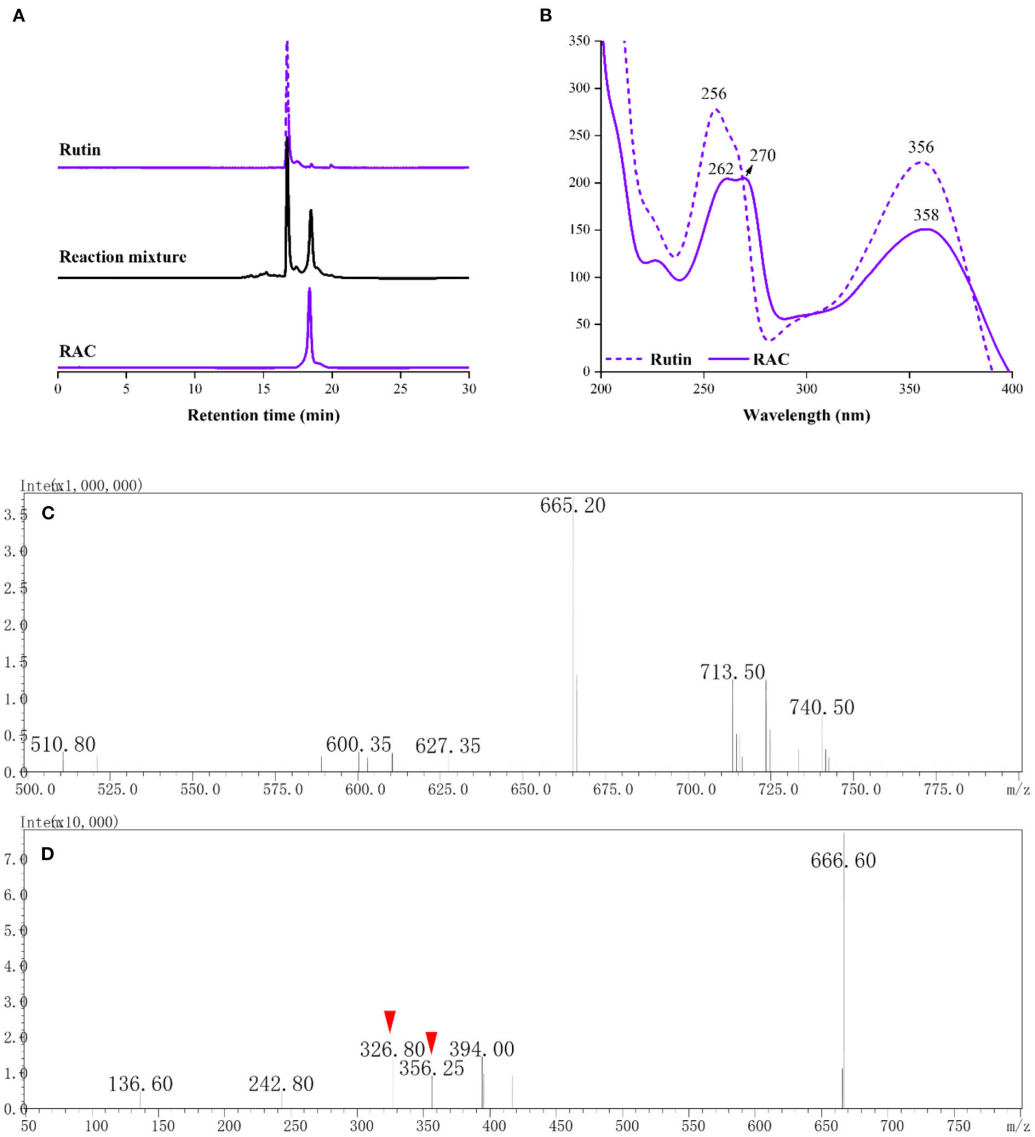
The **(A)** HPLC chromatograms and corresponding **(B)** UV spectra of rutin, reaction mixture and RAC, and the **(C)** MS and **(D)** MS/MS spectra of RAC.

Compared with rutin [retention time (*R*_t_), 16.73 min], a newly occurred chromatographic peak (*R*_t_ = 18.46 min) could be seen after 0.5 h of reaction (Figure 2A). This compound was obtained after purification with a chromatographic purity of 99%, and given RAC. As displayed in Figure 2B, it had absorption peaks at 262, 270, and 358 nm, redshifted relative to rutin (absorption peaks at 256 and 356 nm). The molecular weight of RAC was collected prior to the NMR experiment. In the MS of RAC (Figure 2C), a major ion peak [M – H]^−^ at *m/z* 665 was observed, which was 56 mass units (the molecular weight of ACR) higher than rutin [(M – H)^−^: 609]. This deprotonated precursor ion was further subjected to MS/MS analysis (Figure 2D), and the product ion at *m/z* 356 suggested the loss of the rutinoside moiety, whereas the product ion at *m/z* 326 indicated the further loss of [-CO-2H]^−^ moiety.

**Figure 3:**
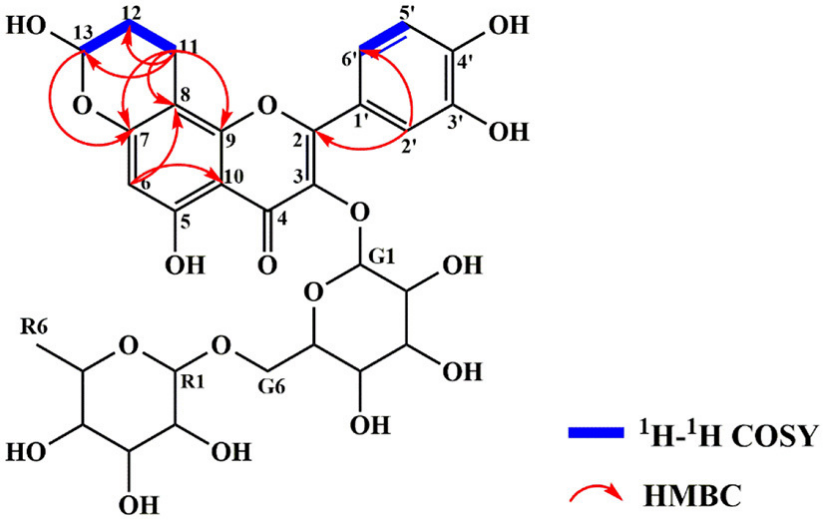
The chemical structure and key ^1^H-^1^H COSY and HMBC correlations of RAC.

The exact structure of RAC was determined by its NMR data. Compared with rutin, the ^1^H NMR spectrum (Supplementary Figure S1) of RAC displayed part of the same resonances, including one methyl at δ_H_ 0.96 (3H, dd, *J* = 15.5, 6.2 Hz), two methines at δ_H_ 4.37 (1H, d, *J* = 1.9 Hz) and 5.35 (1H, d, *J* = 7.6 Hz), and four aromatic protons at δ_H_ 6.17 (1H, d, *J* = 3.7 Hz), 6.90 (1H, d, *J* = 8.5 Hz), 7.62 (1H, d, *J* = 8.5 Hz), and 7.65 (1H, d, *J* = 2.1 Hz). However, the signal at δ_H_ 6.38 was absent, indicating the replacement of proton happened to C-8. In addition, three additional groups of signals, interpreted with the aid of an HSQC experiment (Supplementary Figure S4), suggested the existence of one methine [δ_H_ 5.56 (1H, s)] and two methylene groups [δ_H_ 1.92 (2H, m), 2.85 (2H, m)]. The ^13^C and DEPT 135 data of RAC (Supplementary Figures S2, S3) revealed thirty carbon resonances, including one carbonyl, ten quaternary carbons, fifteen methine groups, three methylene groups, and one methyl. Further comparing with the carbon resonances of rutin, changes in the chemical shift of C-7 and C-8 as well as the appearance of three new signals (δ_C_ 92.9, 26.4 and 14.5) could be observed. The detailed assignments of ^1^H and ^13^C signals of RAC were present in Table 1. Since the 1D NMR data of rutin and RAC exhibited high similarity, comprehensive interpretation of the 2D NMR data of RAC mainly focused on the newly occurred signals. Its ^1^H-^1^H COSY spectrum (Supplementary Figure S5) showed the correlations from δ_H_ 5.56 (H-13) to δ_H_ 1.92 (H-12) and from δ_H_ 1.92 (H-12) to δ_H_ 2.85 (H-11), together with the HMBC (Supplementary Figure S6) correlations from H-11 to C-12/C-13, indicating the fragment of C(13)–C(12)–C(11). Additionally, HMBC correlations from H-11 to C-8/C-9/C-7 indicated the linkage of C-11 to C-8, and from H-13 to C-7 indicated the fragment of C(13)–O–C(7). Therefore, the chemical structure of RAC was established, as shown in Figure 3, which was a novel compound.

**Table 1:** The ^1^H and ^13^C assignments of RAC^a^.

**No**.	**^1^H**	**^13^C**	**^1^H-^1^H COSY**	**HMBC**
2	-	156.5		
3	-	133.5		
4	-	177.6		
5	-	158.6		
6	6.17 (d, *J* = 3.7 Hz, 1H)	99.0		C-5, 7, 8, 10
7	-	158.5		
8	-	101.0		
9	-	152.9		
10	-	104.7		
11	2.85, m, 2H	14.5	H-12	C-7, 8, 9, 12, 13
12	1.92, m, 2H	26.4		C-13
13	5.56, s, 1H	92.9	H-12	C-7
1'	-	121.3		
2'	7.65 (d, *J* = 2.1 Hz, 1H)	115.5		C-2, 3', 4', 6'
3'	-	144.9		
4'	-	148.6		
5'	6.90 (d, *J* = 8.5 Hz, 1H)	116.2		C-3', 4', 6'
6'	7.62 (d, *J* = 8.5 Hz, 1H)	121.6	H-5'	C-2, 2', 4', 5'
G1^b^	5.35 (d, *J* = 7.6 Hz, 1H)	101.1		
G2	3.06–3.69	74.1		
G3		76.5		
G4		70.0		
G5		75.9		
G6		66.9		
R1^b^	4.37 (d, *J* = 1.9 Hz, 1H)	100.6		
R2	3.06–3.69	70.4		
R3		70.6		
R4		71.8		
R5		68.2		
R6	0.95 (dd, *J* = 15.5, 6.2 Hz, 2H)	17.7		

a Measured at 600 (^1^H) and 150 (^13^C) MHz in DMSO-d_6_ for RAC, and chemical shifts were expressed in parts per million (ppm).

b “G” is the abbreviation of “glucose” and “R” of “rhamnose”.

**Figure 4:**
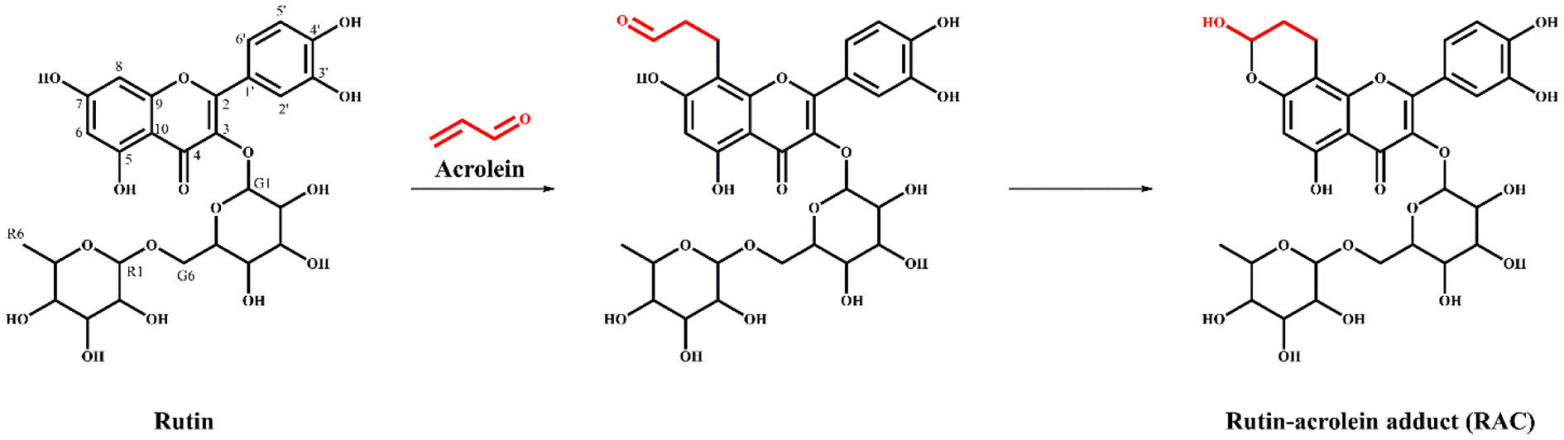
The proposed reaction mechanism of acrolein and rutin to form the hemiacetal adduct RAC.

The reaction mechanism of ACR and polyphenols with *meta*-phenol structures to form the corresponding hemiacetals had been put forward previously (20, 26). Therefore, the chemical rationale for the interaction between ACR and rutin was proposed as followed (Figure 4) the C = C of ACR adducted to C-8 of rutin through electrophilic addition, followed by intramolecular nucleophilic addition between the contiguous hydroxyl at C-7 and the -CHO of ACR to form a more stable cyclic hemiacetal structure.

### Simulated *in vitro* digestion

**Figure 5:**
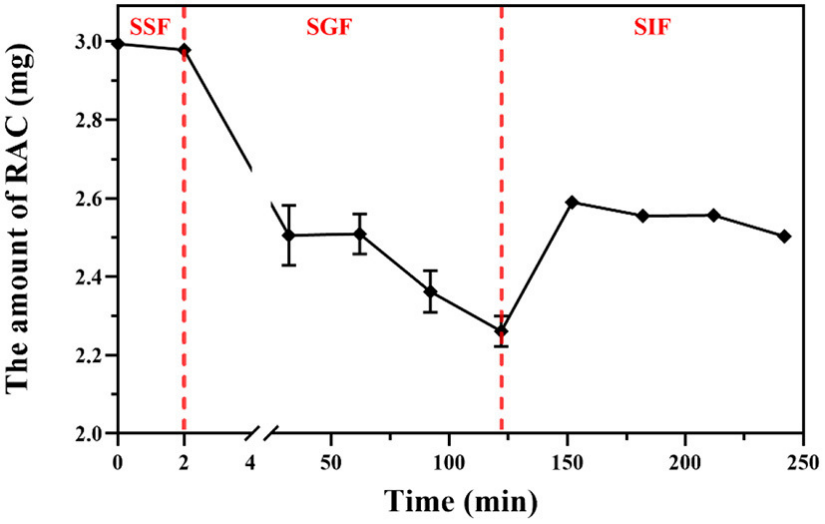
The amount of residual RAC during simulated oral (SSF), gastric (SGF), and intestinal (SIF) digestion *in vitro*.

The content of RAC was calculated based on its calibration curve (*y* = 34274493*x*- 100977; *r*^2^ = 0.9998; *x* was the concentration of each sample and *y* was the corresponding peak area). As depicted in Figure 5 and Supplementary Table S1, after mimic oral digestion, the content of RAC was 0.595 ± 0.0018 mg/mL, and a decrease of 0.33% indicated that RAC remained unchanged at this stage (*p* > 0.05). When the adduct was further subjected to simulated gastric digestion for 120 min, a reduction of 24.41% was observed, from 0.167 ± 0.0042 mg/mL to 0.151 ± 0.0021 mg/mL. Surprisingly, a significant increase in the amount of RAC after 30 min of mimic intestinal digestion was observed, compared with that of RAC after 120 min of mimic gastric digestion (*p* < 0.001). In addition, the amount of RAC after 120 min of mimic intestinal digestion (2.50 ± 0.01 mg) and that of RAC after 30 min of mimic gastric digestion (2.50 ± 0.06 mg) were almost at the same level (*p* > 0.05). In a whole, 83.61% of RAC was remained after *in vitro* simulated oral, gastric, and intestinal digestion.

It was reported that hemiacetals were stable under alkaline conditions, but could be reversibly hydrolyzed back to the starting aldehyde and alcohol in acidic aqueous solution (44, 45). Therefore, the negligible hydrolysis of RAC during simulated oral and intestinal digestion was attributed to the weakly acidic environment (pH 6.8) (*p* > 0.05), while the low pH condition (pH 1.5) during simulated gastric digestion led to the sharp decline of RAC. Furthermore, after 30 min of simulated gastric digestion (Supplementary Figure S7), new peaks could be observed by HPLC analysis, and the peak areas increased with time. However, owing to the low content of the newly occurred peaks, their detailed information was hard to obtain in this study. But based on their UV spectra, it was tentatively presumed that they had a similar structure to RAC, with absorbances at 260, 270, and 359 nm.

### Cytotoxicity of the adduct against Caco-2 and GES-1 cells with acrolein as the control

**Figure 6:**
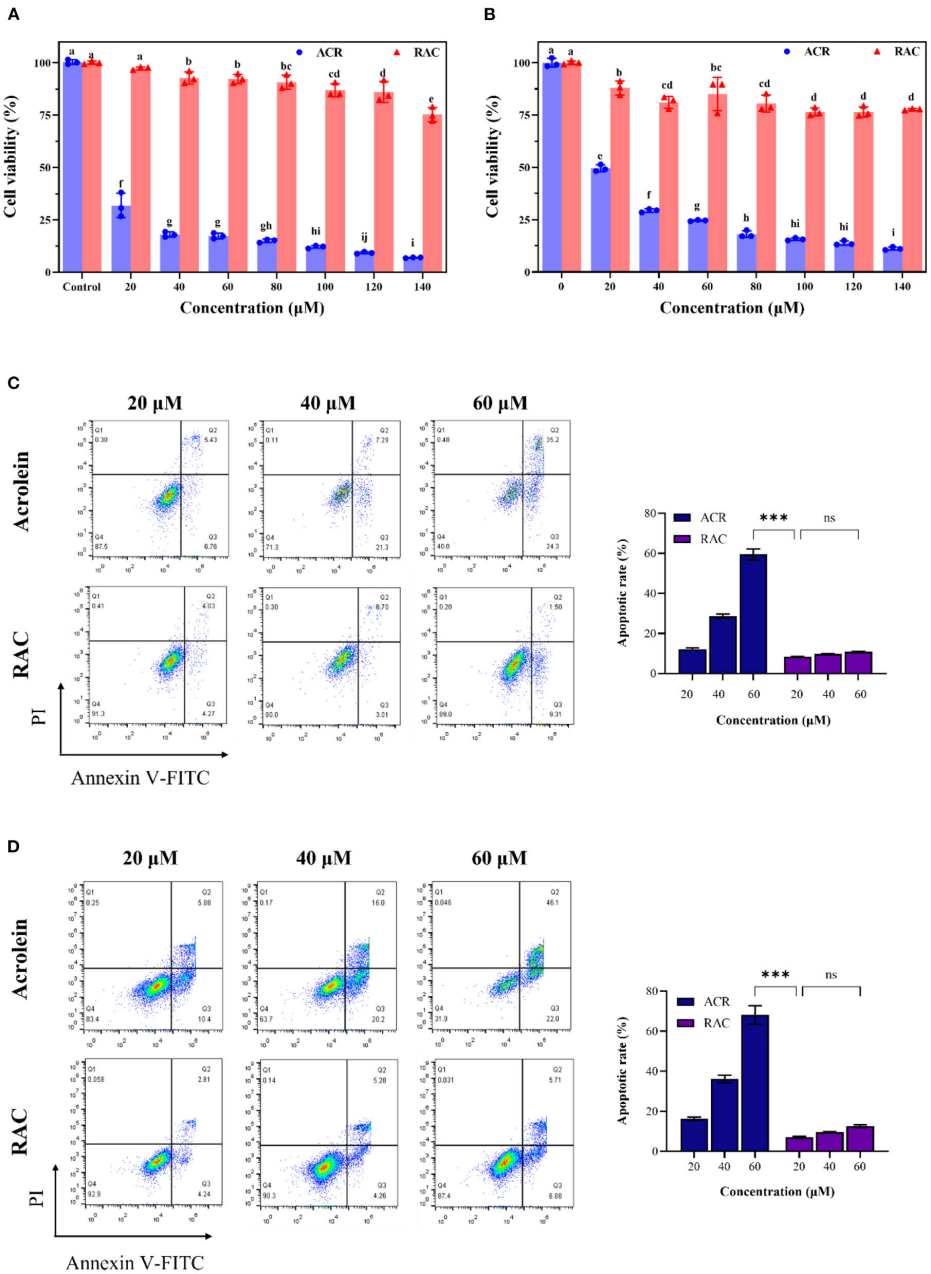
The cell viability of **(A)** Caco-2 and **(B)** GES-1 cells after incubation with ACR (acrolein) and RAC at the concentrations of 20–140 μM for 48 h, and the apoptosis induced by ACR and RAC at the concentrations of 20–60 μM in **(C)** Caco-2 and **(D)** GES-1 cells. Different letters indicated significant differences (*p* < 0.05). ***: *p* < 0.001; ns: not significant.

Given that ingested ACR mainly affected the gastrointestinal system (46), the cytotoxicity of ACR and RAC against GES-1 and Caco-2 cell lines was examined. The CCK-8 assay was one of the effective methods to quantify viable cells, as the formation of orange and water-soluble formazan was positively correlated with the number of living cells (47). As demonstrated in Figures 6A,B, ACR inhibited the cell viability of both Caco-2 and GES-1 cells in a concentration-dependent manner. Specifically, after incubation with ACR for 24 h, the viable cell number of Caco-2 cells decreased by 93.06%, from 31.83% (20 μM) to 6.97% (140 μM), and that of GES-1 cells decreased by 88.78%, from 49.57% (20 μM) to 11.22% (140 μM). In comparison, RAC-induced cell death was relatively modest. More than 75% of both Caco-2 and GES-1 cells survived after exposure to 140 μM of RAC for the same duration. It was also observed that the viability of Caco-2 and GES-1 cells treated with RAC was 10- and 6.5-fold higher than that of ACR-treated Caco-2 and GES-1 cells respectively. Furthermore, the IC_50_ values of ACR for Caco-2 and GES-1 cells were both below 20 μM, while that of RAC for both cells were above 140 μM.

Annexin V had high affinity and specificity for phosphatidylserine, which could appear on the outer side of plasma membrane as a signal of apoptosis, while PI was capable of permeating incomplete membrane and binding to DNA (48). Therefore, Annexin V and PI double staining was further introduced to determine apoptosis. It could be seen that ACR concentration-dependently induced apoptosis in Caco-2 and GES-1 cells as well (Figures 6C,D). After Caco-2 cells were treated with 20, 40, and 60 μM ACR for 24 h, the number of viable cells declined to 87.5, 71.3, and 40.0%, respectively, while that of apoptotic cells increased to 12.19, 28.59, and 59.50%, respectively. In contrast, Caco-2 cells treated with different concentrations of RAC exhibited a negligible increase in apoptosis (*p* > 0.05), from 8.3% (20 μM) to 10.81% (60 μM). Furthermore, the apoptotic rate of cells exposed to 60 μM of ACR was 5.5 times higher than that of the same concentration of RAC. The above data demonstrated that ACR-induced apoptosis was significantly alleviated after ACR was captured by rutin to form RAC. Similar results could be seen in GES-1 cells. To be specific, the apoptotic rate of ACR-treated GES-1 cells increased from 16.28% (20 μM) to 68.10% (60 μM). However, even exposure to 60 μM of RAC caused only 12.59% of GES-1 cells undergoing apoptosis, 5.4-fold <60 μM ACR-treated group. Consequently, it could be concluded that the formation of RAC attenuated ACR-induced cytotoxicity against Caco-2 and GES-1 cells.

### Determination of MMP in Caco-2 and GES-1 cells

The cytotoxic effects of ACR against Caco-2 and GES-1 cells were also studied in other literature, and there was accumulating evidence suggesting the link between mitochondrial dysfunction and apoptosis (9, 49). Hence, changes of MMP in cells were further measured by JC-1, as the decrease of MMP in cells was an iconic issue found in early apoptotic cells (50). JC-1 was a lipophilic fluorescent probe and was capable of entering the mitochondrial matrix, where it aggregated to form polymers with red fluorescence under high membrane potential while existed as monomers with green fluorescence under low membrane potential. In addition, the enhancement of green fluorescence along with the reduction of red fluorescence was correlated with the loss of MMP (51).

**Figure 7:**
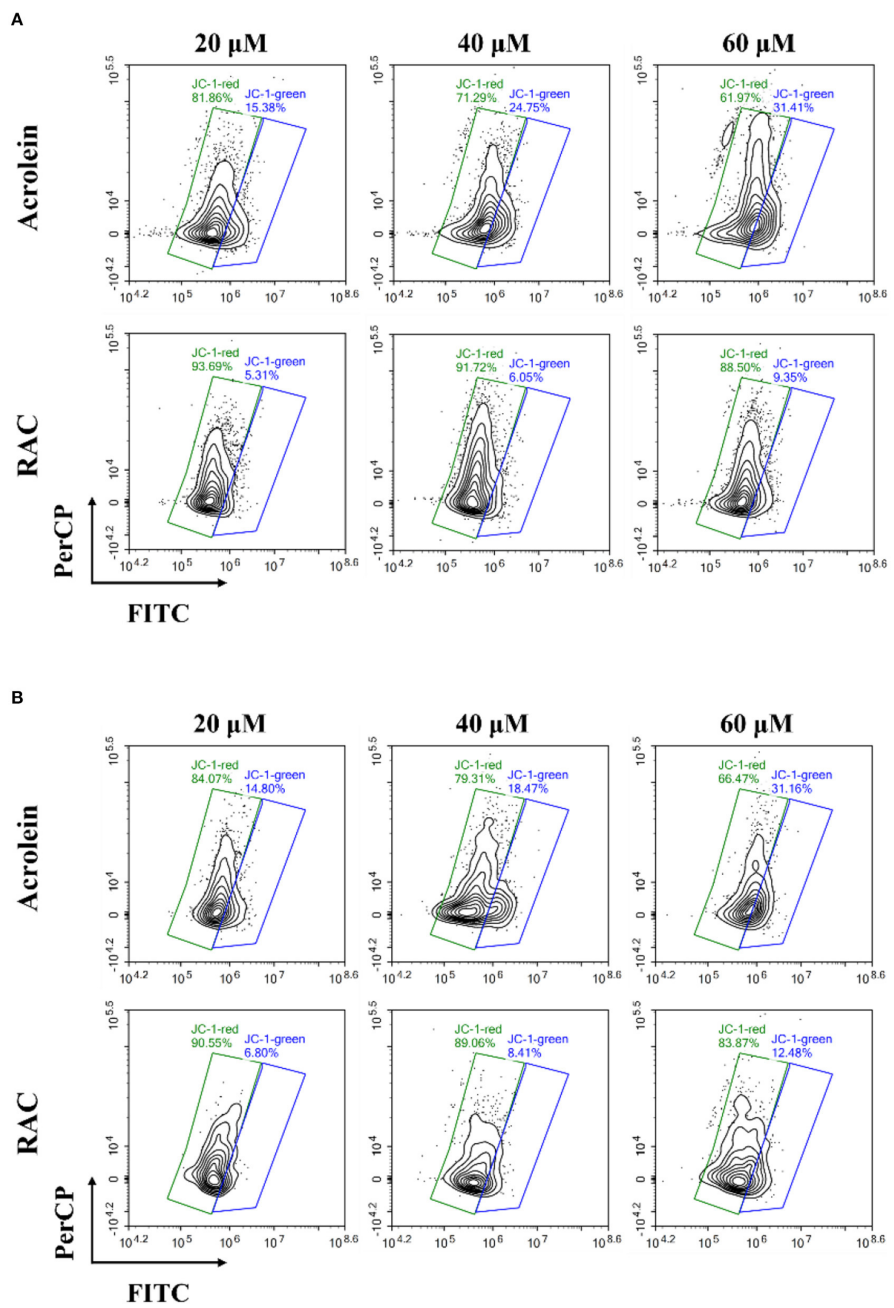
The changes of the mitochondrial membrane potential (MMP) in **(A)** Caco-2 and **(B)** GES-1 cells after incubation with acrolein and RAC at the concentrations of 20, 40, and 60 μM respectively.

As depicted in Figure 7A, ACR induced the loss of MMP in Caco-2 cells dose-dependently, with the increase of green fluorescence and the decrease of red fluorescence. And the ratio of red/green fluorescence intensity in ACR-treated group significantly decreased from 26.54 (control, data not shown) to 1.97 (60 μM) (Supplementary Table S2) (*p* < 0.001), highly suggesting depolarization of the MMP in Caco-2 cells. However, RAC attenuated the transformation from red to green fluorescence (Figure 7A), and the ratio of red/green fluorescence intensity of the 60 μM RAC-treated group (9.47) was even higher than that of the 20 μM ACR-treated group (5.32) (Supplementary Table S2). Similar phenomenon was also observed in GES-1 cells incubated with ACR and RAC (Figure 7B). With the increased concentrations of ACR, the red fluorescence was reduced while the green fluorescence was increased. Besides, the ratio of red/green fluorescence intensity in ACR-treated group significantly reduced from 17.71 (control, data not shown) to 2.13 (60 μM) (*p* < 0.001), further indicating the disruption of MMP in GES-1 cells (Supplementary Table S3). However, the reduction of MMP in GES-1 cells was alleviated when incubated with RAC (Figure 7B), where the ratio of red/green fluorescence intensity of 60 μM RAC-treated group (6.72) was also higher than that of 20 μM ACR-treated group (5.68) (Supplementary Table S3). As a result, it could be tentatively concluded that RAC attenuated ACR-induced cytotoxicity in Caco-2 and GES-1 cells by ameliorating the loss of MMP in mitochondria.

## Conclusion

ACR was not only a foodborne pollutant but also a trigger of several serious diseases. In this study, the capacity of rutin, a nutrient polyphenol found in several frequently consumed ingredients like onions and peppers, to scavenge ACR was investigated. It was shown that rutin could scavenge more than 98% of ACR under the set conditions (temperature, 80°C; reaction time, 2 h; and the molar ratio of rutin/ACR, 2/1). Besides, the results indicated that rutin scavenged ACR through the formation of a hemiacetal adduct (RAC), of which the structure was identified for the first time, and C-8 and the hydroxyl at C-7 of rutin were the reaction sites for ACR. Due to the presence of the hemiacetal structure that was pH-sensitive, RAC was partially degraded after the three-stage simulated digestion, with 83.61% remained. Furthermore, the results revealed that RAC ameliorated ACR-induced cytotoxicity against Caco-2 and GES-1 cells through improvement of the loss of MMP. Overall, the above observations suggested that rutin could also be one of the potential ACR scavengers, and rutin-enriched dietary materials might contribute to limit ACR released during domestic cooking.

## Data availability statement

The original contributions presented in the study are included in the article/Supplementary materials, further inquiries can be directed to the corresponding authors.

## Author contributions

PC: methodology and writing-original draft. SL: methodology and investigation. ZY and PL: data curation. CW: supervision. HZ: resources. YL: visualization. SO: conceptualization. GL: conceptualization, review, and funding acquisition. All authors contributed to the article and approved the submitted version.

## Funding

This work was supported by grants from Guangdong Basic and Applied Basic Research Foundation (2019A1515011967 and 2021A1515110430), Doctoral Workstation Foundation of Guangdong Second Provincial General Hospital (2021BSGZ017), Science and Technology Planning Project of Guangdong Province (202002030404 and 2021B1212030008), Foundation of Guangdong Second Provincial General Hospital (3DB2020014 and TJGC-2021011).

## Conflict of interest

The authors declare that the research was conducted in the absence of any commercial or financial relationships that could be construed as a potential conflict of interest.

## Publisher's note

All claims expressed in this article are solely those of the authors and do not necessarily represent those of their affiliated organizations, or those of the publisher, the editors and the reviewers. Any product that may be evaluated in this article, or claim that may be made by its manufacturer, is not guaranteed or endorsed by the publisher.
