# Supplementary material for: Rutin alleviated acrolein-induced cytotoxicity in Caco-2 and GES-1 cells by forming a cyclic hemiacetal product
Source: Front Nutr. 2022 Aug 16;9:976400. doi: 10.3389/fnut.2022.976400 (PMC9424909; doi:10.3389/fnut.2022.976400)
Supplement: Supplementary file 1 [file Data_Sheet_1.docx]

Supplementary Material

Supplementary Figure Captions

**Figure S1.** The ^1^H NMR spectrum of RAC.

**Figure S2.** The ^13^C NMR spectrum of RAC.

**Figure S3.** The DEPT 135 spectrum of RAC.

**Figure S4.** The HSQC spectrum of RAC.

**Figure S5.** The ^1^H-^1^H COSY spectrum of RAC.

**Figure S6.** The HMBC spectrum of RAC.

**Figure S7.** The chromatograms of simulated gastric digestion at different durations. Compared with the control, the peak areas of the two peaks with the retention times of 22.240 and 22.563 min increased with the time.

**Supplementary Figures**

**
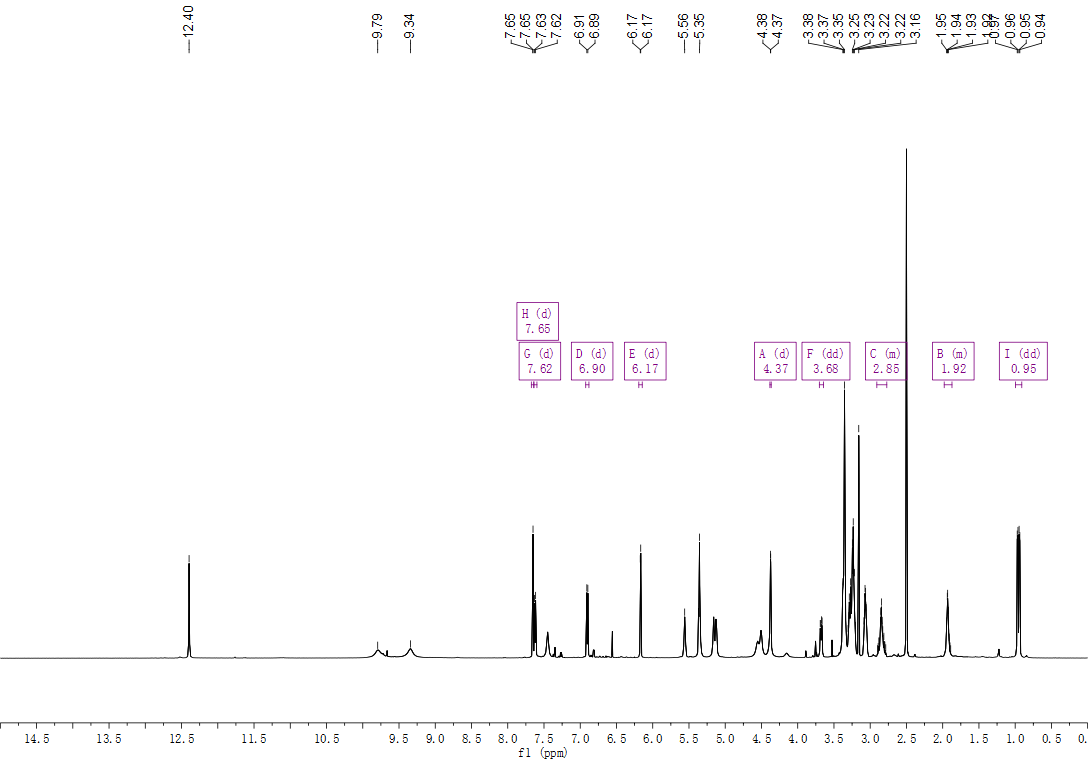
**

**Supplementary Figure 1.** The ^1^H NMR spectrum of RAC.

**
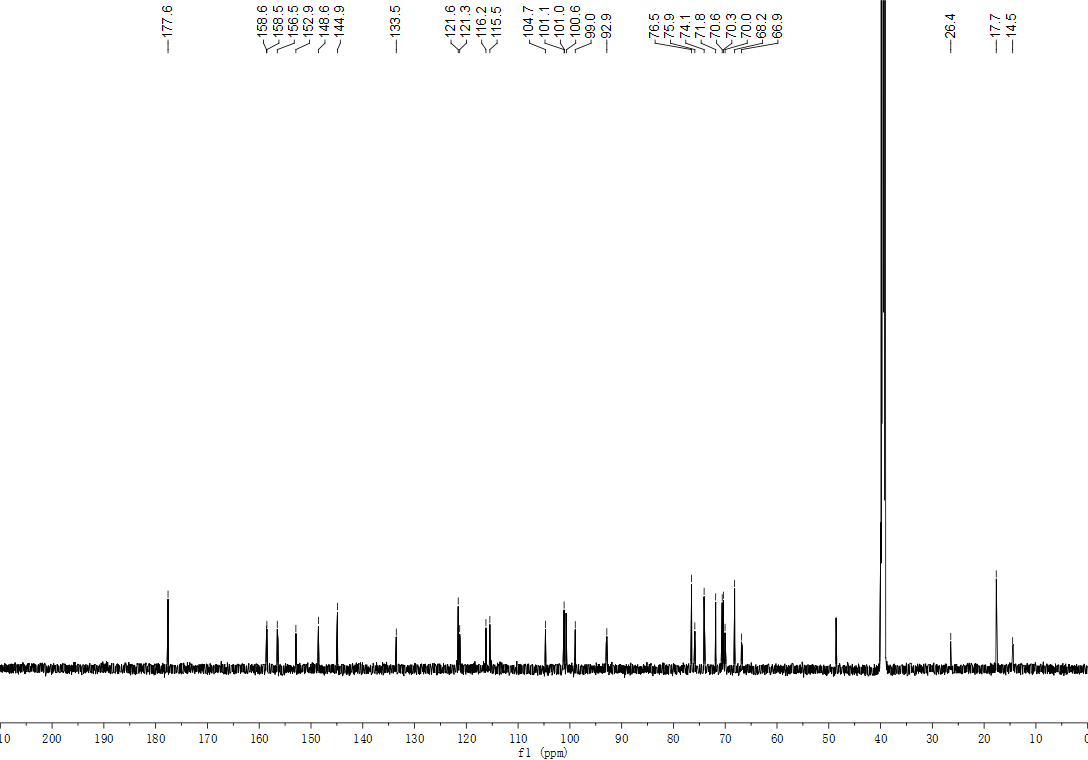
**

**Supplementary Figure 2.** The ^13^C NMR spectrum of RAC.

**
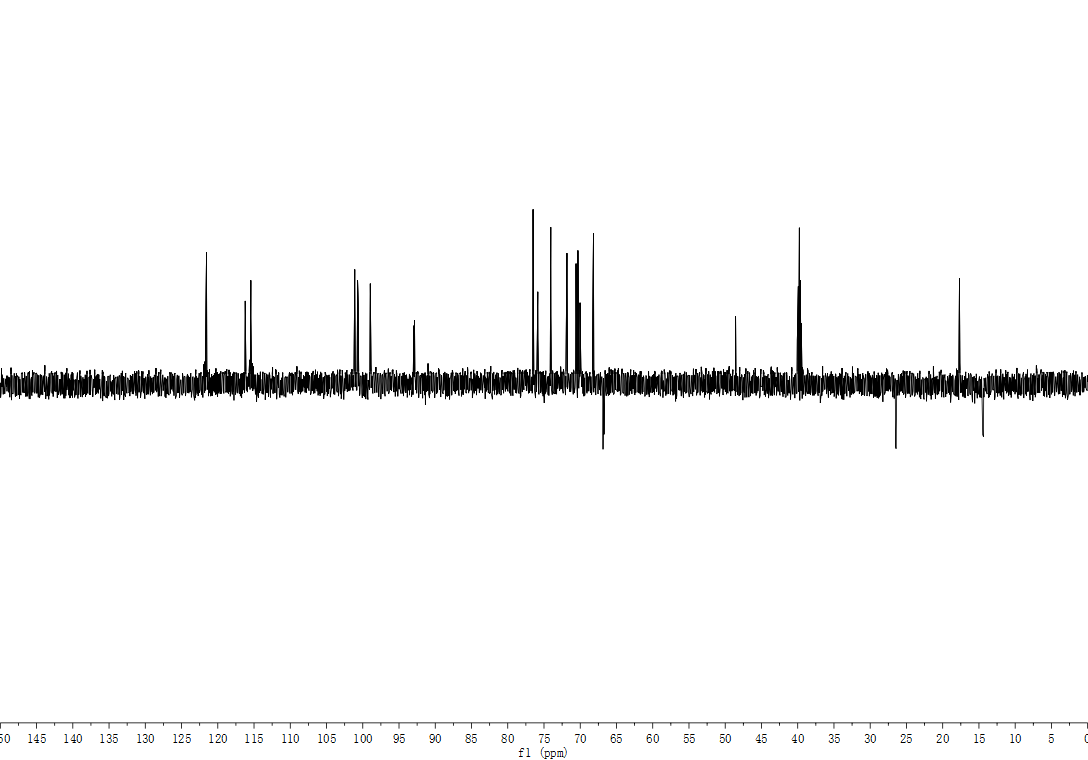
**

**Supplementary Figure 3.** The DEPT 135 spectrum of RAC.

**
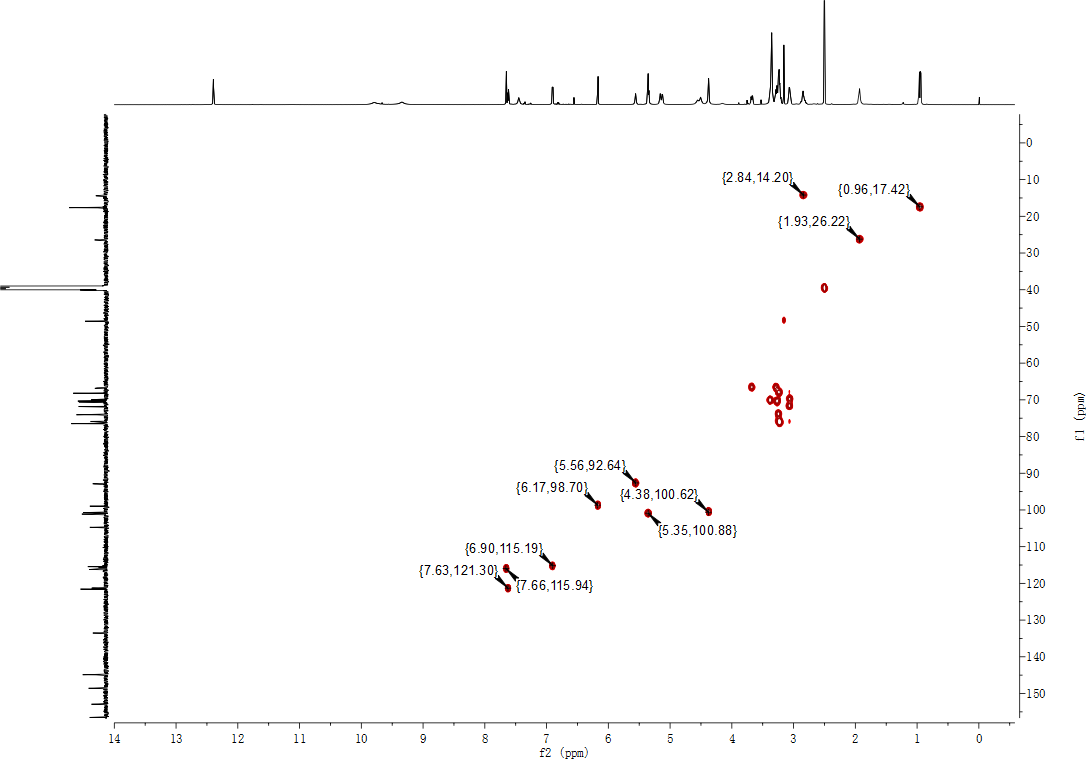
**

**Supplementary Figure 4.** The HSQC spectrum of RAC.

**Supplementary Figure 5.** The ^1^H-^1^H COSY spectrum of RAC.

**Supplementary Figure 6.** The HMBC spectrum of RAC.

**Supplementary Figure 7.** The chromatograms of simulated gastric digestion at different durations. Compared with the control, the peak areas of the two peaks with the retention times of 22.240 and 22.563 min increased with the time.

Supplementary Table Captions

**Table S1.** The content and amount of RAC after simulated oral (SSF), gastric (SGF), and intestinal digestion (SIF). Results were shown as mean ± standard deviation (SD), and different letters indicated significant differences (*p* < 0.05).

**Table S2.** The ratio of red/green fluorescence intensity of Caco-2 cells incubated with different concentrations of acrolein and RAC. Results were shown as mean ± SD.

**Table S3.** The ratio of red/green fluorescence intensity of GES-1 cells incubated with different concentrations of acrolein and RAC**.** Results were shown as mean ± SD.

**Supplementary Tables**

**Supplementary Table 1.** The content and amount of RAC after simulated oral (SSF), gastric (SGF), and intestinal digestion (SIF)^a^.

| Stages | The content of RAC (mg/mL) | The amount of RAC (mg) | Decrease (%) |
| --- | --- | --- | --- |
| Initial | 0.599 ± 0.0017 | 2.99 ± 0.01 |  |
| SSF |  |  |  |
| 2 min | 0.595 ± 0.0018 | 2.98 ± 0.01 | 0.33 |
| SGF |  |  |  |
| 30 min | 0.167 ± 0.0042 | 2.50 ± 0.06 a | 16.39 |
| 60 min | 0.167 ± 0.0028 | 2.51 ± 0.04 a | 16.05 |
| 90 min | 0.157 ± 0.0029 | 2.36 ± 0.04 b | 21.07 |
| 120 min | 0.151 ±0.0021 | 2.26 ± 0.03 b | 24.41 |
| SIF |  |  |  |
| 30 min | 0.074 ± 0.0002 | 2.59 ± 0.01 a | 13.38 |
| 60 min | 0.073 ± 0.0002 | 2.55 ± 0.01 b | 14.72 |
| 90 min | 0.073 ± 0.0002 | 2.56 ± 0.01 b | 14.38 |
| 120 min | 0.072 ± 0.0004 | 2.50 ± 0.01 c | 16.39 |
| ^a^ Results were shown as mean ± standard deviation (SD), and different letters indicated significant differences (*p* < 0.05). | | | |

**Supplementary Table 2.** The ratio of red/green fluorescence intensity of Caco-2 cells incubated with different concentrations of acrolein and RAC ^a^.

| Red/green fluorescence intensity | Acrolein | RAC |
| --- | --- | --- |
| Control | 26.54 ± 0.87 | |
| 20 μM | 5.32 ± 0.17 | 17.64 ± 0.69 |
| 40 μM | 2.88 ± 0.09 | 15.16 ± 0.59 |
| 60 μM | 1.97 ± 0.06 | 9.47 ± 0.37 |
| ^a^ Results were shown as mean ± SD. | | |

**Supplementary Table 3.** The ratio of red/green fluorescence intensity of GES-1 cells incubated with different concentrations of acrolein and RAC ^a^.

| Red/green fluorescence intensity | Acrolein | RAC |
| --- | --- | --- |
| Control | 17.71 ± 0.97 | |
| 20 μM | 5.68 ± 0.24 | 13.32 ± 0.43 |
| 40 μM | 4.29 ± 0.17 | 10.59 ± 0.33 |
| 60 μM | 2.13 ± 0.10 | 6.72 ± 0.30 |
| ^a^ Results were shown as mean ± SD. |  |  |
